# Evaluation of the default-mode network by quantitative ^15^O-PET: comparative study between cerebral blood flow and oxygen consumption

**DOI:** 10.1007/s12149-018-1272-x

**Published:** 2018-06-22

**Authors:** Jo Aoe, Tadashi Watabe, Eku Shimosegawa, Hiroki Kato, Yasukazu Kanai, Sadahiro Naka, Keiko Matsunaga, Kayako Isohashi, Mitsuaki Tatsumi, Jun Hatazawa

**Affiliations:** 1 Department of Nuclear Medicine and Tracer Kinetics, Osaka University Graduate School of Medicine, 2-2 Yamadaoka, Suita, Osaka 565-0871 Japan; 2 Department of Molecular Imaging in Medicine, Osaka University Graduate School of Medicine, 2-2 Yamadaoka, Suita, Osaka 565-0871 Japan; 3 Department of Pharmacology, Osaka University Hospital, 2-15 Yamadaoka, Suita, Osaka 565-0871 Japan; 4 Department of Radiology, Osaka University Hospital, 2-15 Yamadaoka, Suita, Osaka 565-0871 Japan

**Keywords:** Default-mode network, Functional correlation, ^15^O PET, Cerebral blood flow, Cerebral metabolic rate of oxygen

## Abstract

**Objective:**

Resting-state functional MRI (rs-fMRI) has revealed the existence of a default-mode network (DMN) based on spontaneous oscillations of the blood oxygenation level-dependent (BOLD) signal. The BOLD signal reflects the deoxyhemoglobin concentration, which depends on the relationship between the regional cerebral blood flow (CBF) and the cerebral metabolic rate of oxygen (CMRO_2_). However, these two factors cannot be separated in BOLD rs-fMRI. In this study, we attempted to estimate the functional correlations in the DMN by means of quantitative ^15^O-labeled gases and water PET, and to compare the contribution of the CBF and CMRO_2_ to the DMN.

**Methods:**

Nine healthy volunteers (5 men and 4 women; mean age, 47.0 ± 1.2 years) were studied by means of ^15^O-O_2_, ^15^O-CO gases and ^15^O-water PET. Quantitative CBF and CMRO_2_ images were generated by an autoradiographic method and transformed into MNI standardized brain template. Regions of interest were placed on normalized PET images according to the previous rs-fMRI study. For the functional correlation analysis, the intersubject Pearson’s correlation coefficients (*r*) were calculated for all pairs in the brain regions and correlation matrices were obtained for CBF and CMRO_2_, respectively. We defined *r* > 0.7 as a significant positive correlation and compared the correlation matrices of CBF and CMRO_2_.

**Results:**

Significant positive correlations (*r* > 0.7) were observed in 24 pairs of brain regions for the CBF and 22 pairs of brain regions for the CMRO_2_. Among them, 12 overlapping networks were observed between CBF and CMRO_2_. Correlation analysis of CBF led to the detection of more brain networks as compared to that of CMRO_2_, indicating that the CBF can capture the state of the spontaneous activity with a higher sensitivity.

**Conclusions:**

We estimated the functional correlations in the DMN by means of quantitative PET using ^15^O-labeled gases and water. The correlation matrix derived from the CBF revealed a larger number of brain networks as compared to that derived from the CMRO_2_, indicating that contribution to the functional correlation in the DMN is higher in the blood flow more than the oxygen consumption.

## Introduction

The default-mode network (DMN) was discovered by Raichle et al., as a set of brain regions that typically deactivate during performance of cognitive tasks by perfusion PET [1, 2]. Since DMN is active in the resting state, it is reported to have a relationship with our consciousness and has drawn much attention from researchers. Until now, researchers have mainly investigated the DMN by resting state functional MRI (rs-fMRI) based on functional connectivities using the blood oxygenation level dependent (BOLD) signal [3]. For example, Fox et al. discovered 13 foci of DMN using rs-fMRI by calculating functional connectivity between voxels and three seed regions: the medial prefrontal cortex (ventral), the posterior cingulate cortex, and the left lateral parietal cortex [4]. The BOLD signal reflects the deoxyhemoglobin concentration, which depends on the relationship between the regional cerebral blood flow (CBF) and cerebral metabolic rate of oxygen (CMRO_2_). However, these two factors cannot be separated in BOLD rs-fMRI. In this study, we attempted to estimate the functional correlations in the DMN by means of quantitative ^15^O-labeled gases and water PET, and to compare the contribution of CBF and CMRO_2_ to the DMN. While a similar study has been conducted previously using FDG-PET [5], there is still no report of analysis of the CBF and CMRO_2_ to identify DMN correlations. The purpose of this study was to evaluate the functional correlations in the DMN by means of quantitative ^15^O-labeled gases and water PET, and to compare the contribution of the CBF and CMRO_2_ to the DMN.

## Materials and methods

### Participants

^15^O-PET was performed in nine normal volunteers (4 men and 5 women; mean age ± SD = 50.9 ± 0.4 years). The criteria for defining “normal” volunteers were as follows: (1) no past history of neurological and psychiatric disorders, heart failure, liver or renal dysfunction, respiratory diseases, acute inflammatory disease, autoimmune diseases, or cancer, (2) no smoking or alcohol habit, (3) no significant abnormality on MR imaging or MR angiography of the brain, and (4) no history of medication within the previous 3 months [6]. This study was conducted with the approval of the Ethics Committee of Osaka University Hospital. Written informed consent was obtained from all participants.

### PET measurements

PET images were obtained in 3-D mode using the SET-3000 GCT/X scanner (Shimadzu Corp., Kyoto, Japan). The intrinsic spatial resolution was 3.5-mm full-width at half maximum (FWHM) in-plane and 4.2-mm FWHM axially. Subjects were placed lying on the bed of the PET system in a resting state but awake condition with the head fixed. Transmission scanning with a ^137^Cs point source was performed for attenuation correction. The PET images were reconstructed by a filtered-back projection method after 3D Gaussian smoothing with a 6-mm FWHM. Scattered radiation was corrected by the hybrid dual-energy window method combined with a convolution-subtraction method, and estimation of the true scatter-free component of the standard photo peak window was performed on a sonographic basis [7, 8]. The cerebral blood volume (CBV), CMRO_2_, oxygen extraction fraction (OEF), and CBF were measured by means of C^15^O and ^15^O_2_ gas inhalation, and H_2_^15^O injection [8]. A series of ^15^O-gases and ^15^O-water PET scans were performed once per subject. A cannula was inserted into the radial artery for arterial input. CBV measurement was performed with 4-min static scanning after 1 min of continuous inhalation of C^15^O gas (3.0 GBq/min) followed by a 3-min interval [9]. Arterial blood was collected three times during the scanning to measure the whole-blood radioactivity. OEF was measured by 3-min scanning starting simultaneously with 1.5-min ^15^O_2_ gas bolus inhalation (1.0 GBq/min). Continuous arterial blood sampling was performed using a β-detector system to determine the whole-arterial blood radioactivity. CMRO_2_ and OEF were calculated by an autoradiographic method [9–12]. The CBV data were used to correct for intravascular hemoglobin-bound ^15^O_2_ [13]. CBF was measured by 3 min of scanning started simultaneously with the intravenous bolus injection of H_2_^15^O (370 MBq) [10, 14]. Continuous arterial blood sampling was also performed with a β-detector system (Continuous Blood radioactivity sampling System, Shimadzu Corp., Kyoto, Japan). Delay and dispersion occurring in the β-detector system were corrected by the methods described previously [15]. Quantitation of the reconstructed PET images by the 3-D mode PET scanner has been validated in a previous report [8].

### Data analysis

**Fig. 1:**
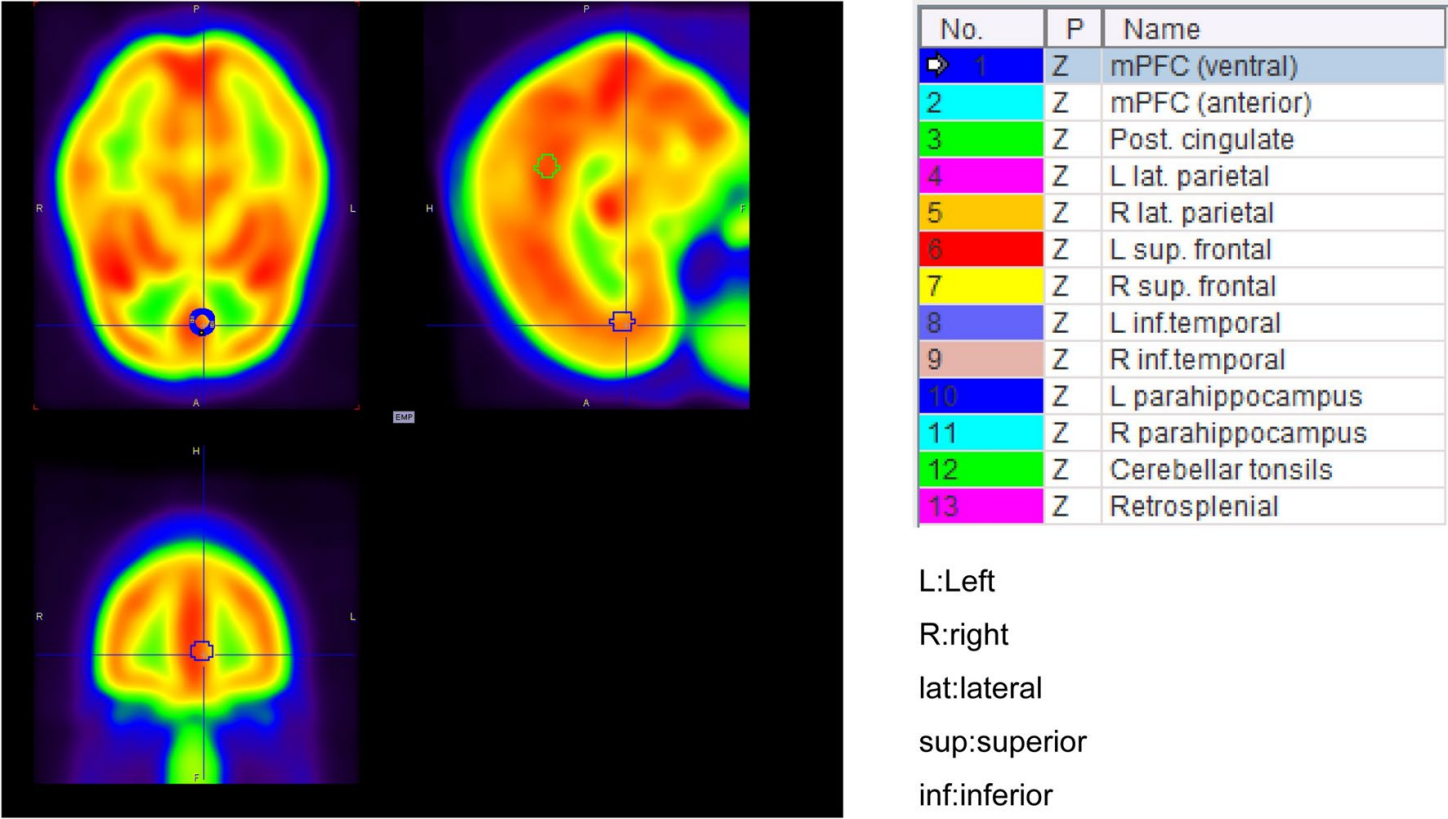
Setting of ROIs using a 12-mm sphere (note that the center of the sphere is located in the specific MNI coordinate point)

We normalized the CBF images to the Montreal Neurological Institute (MNI) standardized brain by applying affine transformation, using International Consortium for Brain Mapping (ICBM) space template, and nonlinear transformations, using the built-in water PET template, by SPM8 (Wellcome Trust Centre for Neuroimaging). For normalization of the CMRO_2_ images, we applied the same transformations as those for the CBF. To calculate the ROI value, we used the PMOD3.6 software (PMOD Technologies LLC). For the ROI of the DMN, we used the same 12-mm spherical ROI as those in the previous rs-fMRI study of the DMN in the MNI coordinates (Fig. 1) [4, 16]. Center locations of the ROIs are shown in Table 1. CBF and CMRO_2_ values of each brain regions were compared by paired t-test with Bonferroni correction and probability values of less than 0.05 divided by the number of pairs (13 combinations of 2) were considered to denote statistical significance.

**Table 1:** Center location of ROIs of DMN used in this study

Regions	Talairach coordinates (*x, y, z*)	MNI coordinates (*x, y, z*)
Medial prefrontal cortex (ventral)	(− 3, 39, − 2)	(− 3, 44, − 2)
Medial prefrontal cortex (anterior)	(1, 54, 21)	(2, 57, 24)
Posterior cingulate cortex	(− 2, − 36, 37)	(− 3, − 39, 39)
Left lateral parietal cortex	(− 47, − 67, 36)	(− 47, − 70, 37)
Right lateral parietal cortex	(53, − 67, 36)	(52, − 71, 38)
Left superior frontal cortex	(− 14, 38, 52)	(− 15, 35, 58)
Right superior frontal cortex	(17, 37, 52)	(17, 34, 57)
Left inferior temporal cortex	(− 61, − 33, −15)	(− 65, − 31, − 22)
Right inferior temporal cortex	(65, − 17, −15)	(69, − 14, − 23)
Left parahippocampal gyrus	(− 22, − 26, −16)	(− 23, − 25, − 24)
Right parahippocampal gyrus	(25, − 26, −14)	(26, − 25, − 21)
Cerebellar tonsils	(7, − 52, −44)	(7, − 50, − 55)
Retro-splenial	(3, − 51, 8)	(3, − 52, 5)

**Fig. 2:**
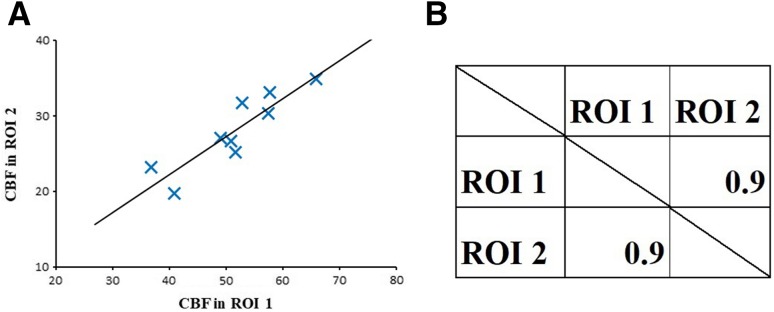
Correlation analysis between pairs of brain regions and correlation matrices. Above example calculated the intersubject Pearson’s correlation coefficient between a pair of brain regions showing a significant positive correlation. Each plot in the left graph represents the data for one subject

For each ROI pair, we calculated the intersubject Pearson’s correlation coefficient (*r*) by plotting the quantitative values of each ROI (nine data points for each pair of brain regions) and obtained a correlation matrix (Fig. 2). *r* > 0.7 was defined as a significant positive correlation. When the number of data is 9, the 95% confidence interval is 0.06703–0.9312 for *r* = 0.7. Therefore, *r* = 0.7 is regarded as a reasonable threshold for determining a significant positive correlation.

We assumed that two brain regions were functionally correlated when there was a significant positive intersubject Pearson’s correlation between them according to the previous study [17]. Calculated functional correlations were visualized using the BrainNet Viewer (Xia et al. [18], http://www.nitrc.org/projects/bnv/) [18].

## Results

The quantitative values of the CBF and CMRO_2_ in various brain regions are summarized in Table 2. The CBF and CMRO_2_ values were 50.4 ± 5.9 mL/100 mL/min and 3.6 ± 0.5 mL/100 mL/min in the medial prefrontal cortex (ventral), 47.8 ± 7.6 mL/100 mL/min and 3.7 ± 0.8 mL/100 mL/min in the posterior cingulate cortex, and 40.2 ± 6.6 mL/100 mL/min and 3.3 ± 0.5 mL/100 mL/min in the left lateral parietal cortex.

**Table 2:** Quantitative values for various brain regions in the nine subjects

	CBF (mL/100 mL/min)	CMRO_2_ (mL/100 mL/min)
vMPFC	50.4 ± 5.9^a,c,d,e,l^	3.6 ± 0.5^a,d,e,g,k,l^
aMPFC^a^	35.8 ± 7.5^d,e,g,k^	2.6 ± 0.5^d,e,g,k^
PCC^b^	47.8 ± 7.6^c,e,f,h,k^	3.7 ± 0.8^e,f,h^
L.LatP ^c^	40.2 ± 6.6 ^d,f,I,j^	3.3 ± 0.5 ^d,f,l^
R.LatP^d^	33.8 ± 6.1^e,j,k^	2.7 ± 0.5^e,k,l^
L.Sup.F^e^	23.5 ± 3.5^f,k^	1.6 ± 0.3^f,i,k^
R.Sup.F^f^	29.3 ± 5.5^k^	2.2 ± 0.4^i,j^
L.IT^g^	39.1 ± 7.9^h^	3.0 ± 0.5^j^
R.IT^h^	37.6 ± 6.7^i^	2.9 ± 0.5^i,l^
L.PHC^i^	36.3 ± 2.8^j,l^	2.4 ± 0.4^j,l^
R.PHC^j^	38.8 ± 4.3	2.4 ± 0.4
Cereb^k^	52.2 ± 9.6^l^	3.6 ± 0.7^l^
Retro-splenial^l^	29.7 ± 7.0	2.0 ± 0.5

*a* anterior, *v* ventral, *L* left, *R* right, *MPFC* medial prefrontal cortex, *Sup.F* superior frontal cortex, *IT* inferior temporal cortex, *PHC* parahippocampal gyrus, *PCC* posterior cingulate cortex, *LatP* lateral parietal cortex, *Cereb* cerebellar tonsils

^a–l^There is a significant difference between the two regions (*p* < 0.05 with Bonferroni correction)

**Fig. 3:**
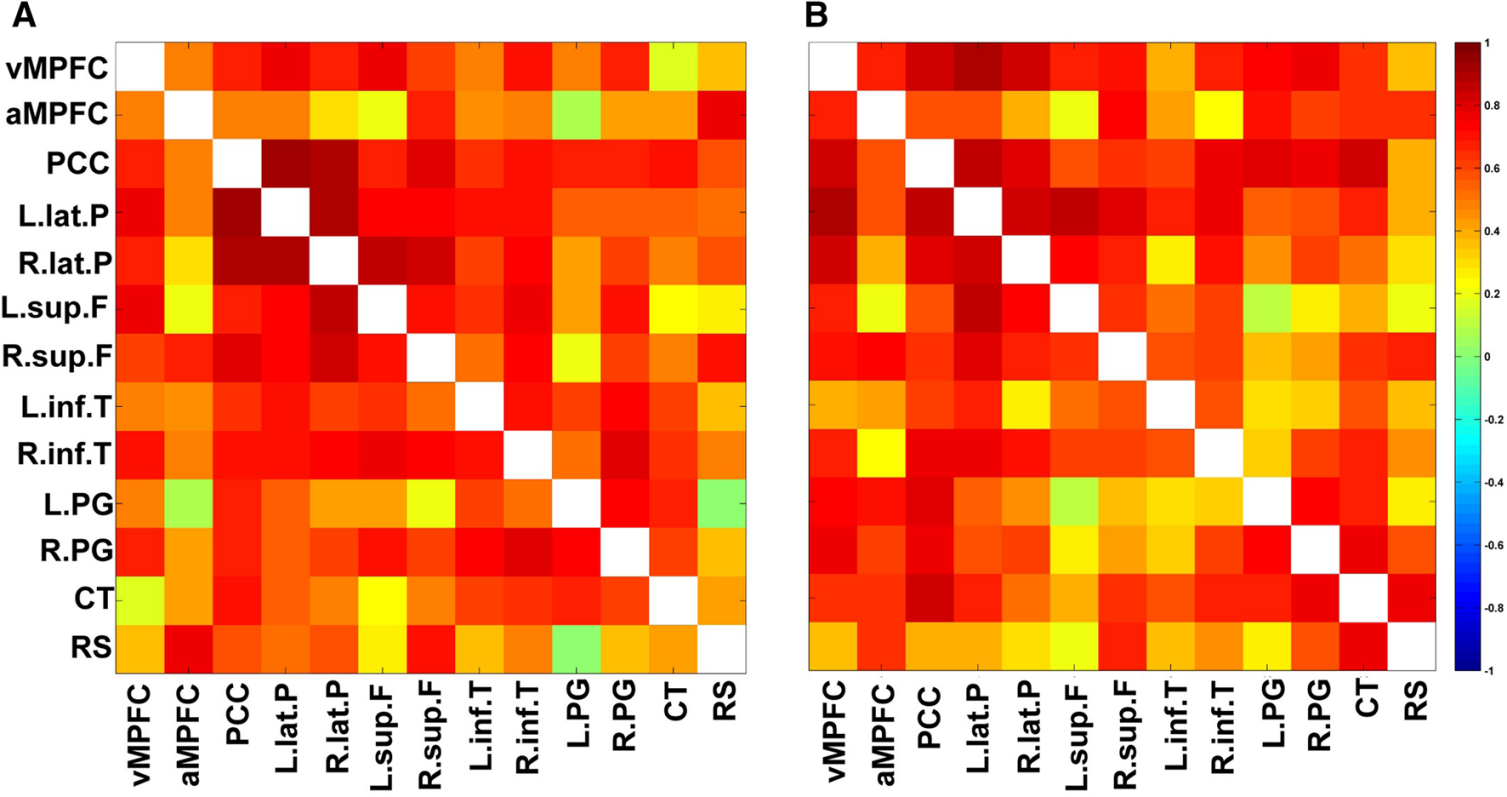
Correlation matrices of the DMN (**a**) based on the CBF (**b**) based on the CMRO_2_. Color represents the value of the correlation coefficients

**Fig. 4:**
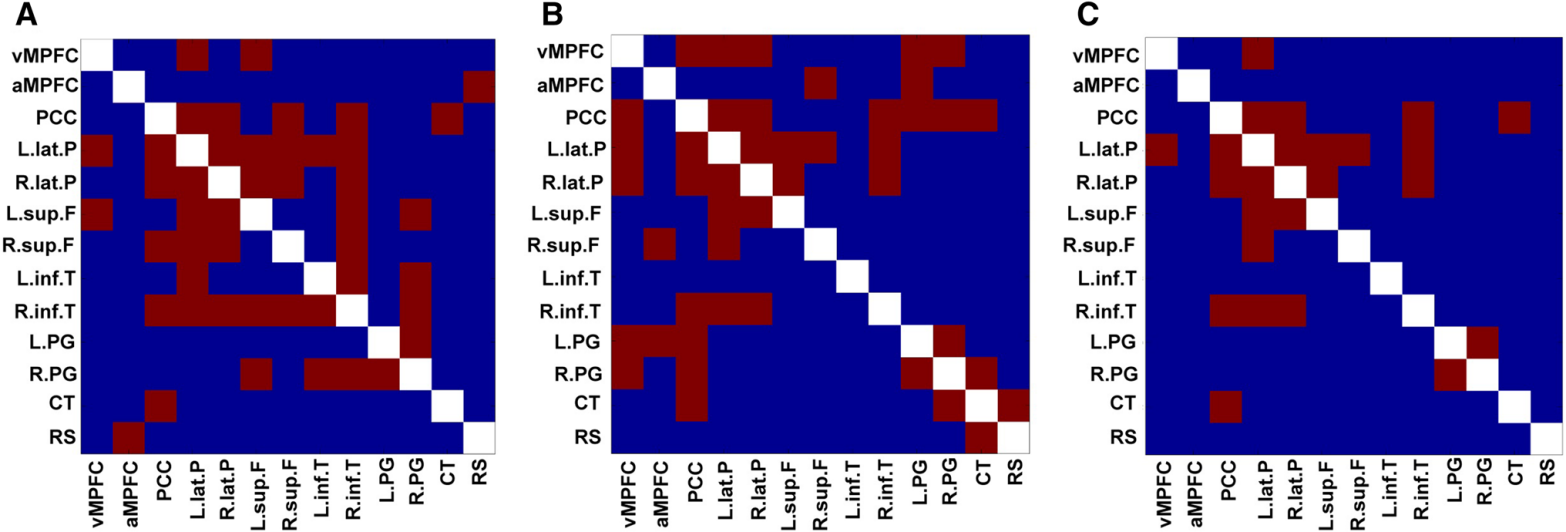
Correlation matrices of the DMN showing significant positive correlations. Red color represents *r* > 0.7 (**a)** based on the CBF (**b**) based on the CMRO_2_ (**c**) overlap between CBF and CMRO_2_

**Fig. 5:**
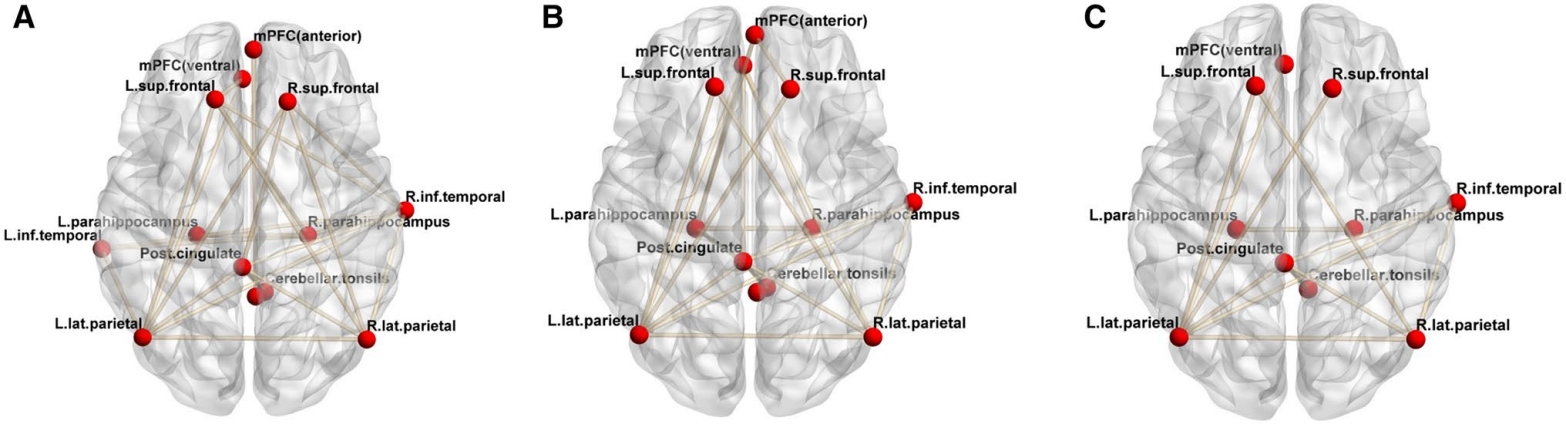
Visualization of the correlation matrices of the DMN showing significant positive correlations. **a** Based on the CBF; **b** CMRO_2_; **c** overlap between CBF and CMRO_2_

The correlation matrices for pairs of DMN ROIs are shown in Fig. 3. In the correlation matrix based on the CBF, the PCC showed significant positive correlations with the left lateral parietal cortex, right lateral parietal cortex, right superior frontal cortex, right inferior temporal cortex, and cerebellar tonsils. The correlation matrix based on the CMRO_2_ showed fewer correlations as compared to that of the CBF. In Fig. 4, 24 pairs of significant positive correlations were observed for the CBF, while 22 pairs were observed for the CMRO_2_. There were 12 overlapping significant positive correlations between the CBF and CMRO_2_. Thus, analysis based on the CBF led to the detection of more brain networks as compared to that based on the CMRO_2_. The significant positive correlations are anatomically illustrated in Fig. 5.

## Discussion

We evaluated the functional correlations of the DMN based on analyses of the CBF and CMRO_2_. More brain networks were found in the analysis based on CBF, and about a half of them overlapped with those identified by analysis of the CMRO_2_. When the local brain activity increases because of physiological fluctuations in the resting state or in response to some stimulation, the local CMRO_2_ increases first, reflecting the increased energy metabolism. Then, the local CBF also increases following the increase of the local CMRO_2_. The extent of increase of the local CBF is usually much larger than that of the local CMRO_2_ [1, 19], which is the main reason for the detection of more brain networks by analysis of the CBF than by analysis of the CMRO_2_. Paulson et al. also reported that CBF and glucose metabolism remain coupled as they increase in proportion during functional activation, whereas oxygen metabolism only increases to a minor degree [20]. Moreover, the CMRO_2_ image is calculated from three scans of H_2_^15^O, ^15^O_2_ and C^15^O, and the long study time for the three scans may blur the regional changes in CMRO_2_, leading to the fewer detection of networks.

We assumed that two brain regions were functionally correlated when there was a significant positive intersubject Pearson’s correlation between them. The underlying idea is that if changes of quantitative CBF or CMRO_2_ values of each subject fluctuate in a similar pattern between the same regions of the brain, we can capture the several different phases of resting state network with significant correlation using the all subject data. A similar method of analysis was also used in the previous study [17].

We used quantitative value itself for the correlation analyses without performing global normalization. Global normalization is used for masking the global fluctuation of blood flow or oxygen metabolism, usually to detect the specific brain region with activation. Since our correlation analysis should include the physiological global fluctuation, we used the quantitative value of CBF and CMRO_2_ directly for the analyses. Nevertheless, future studies should consider global normalization to eliminate the effects of inevitable variations of quantitative values.

We did not compare our results directly with results from rs-fMRI because the number of data and the time scale are significantly different. The rs-fMRI measurement obtained 150 data per each subject, because data were acquired every 2 s over a period of 5 min. On the other hand, our PET measurement obtained one data per each subject because evaluation of the temporal changes was impossible, as every single of the CBF and CMRO_2_ measurements was the average value of 3 min of measurement. In terms of the time scale, rs-fMRI detects changes on the seconds’ scale, whereas PET detects changes on the minutes’ scale, and both data might reflect different brain functions. To evaluate physiological fluctuations in the resting state, we need several repeated scans per subject. Performing several PET scans per subject should be considered in the future study to assess physiological fluctuations for a comparison to the rs-fMRI studies. ^15^O-water PET has several advantages compared to BOLD fMRI, being quantifiable, less deteriorated by movement, and allowing for longitudinal studies [21].

Significant differences were observed between right and left sides in some brain regions (Table 2). Previous study reported the asymmetries in CBF and CMRO_2_, but not in CBV and OEF [22]. In this study, the ratios of CMRO_2_ to CBF were similar between right and left regions, which were consistent with the previous report about OEF.

This study had the limitation of a small number of subjects, so the results could include false-positive signals. Therefore, a further multi-modality study with a sufficient number of subjects should be performed to accurately evaluate the relationships among the CBF, CMRO_2_ and rs-fMRI BOLD signals.

## Conclusions

We estimated the functional correlations in the DMN by means of quantitative PET using ^15^O-labeled gases and water. The correlation matrix derived from the CBF revealed a larger number of brain networks as compared to that derived from the CMRO_2_, indicating that contribution to the functional correlation in the DMN is higher in the blood flow more than the oxygen consumption. A multi-modality study is warranted in the future to evaluate the relationships among the CBF, CMRO_2_ and BOLD signals and establish an integrated system for detection of brain networks.

## References

[1] Raichle ME, MacLeod AM, Snyder AZ, Powers WJ, Gusnard DA, Shulman GL (2001). A default mode of brain function. Proc Natl Acad Sci USA.

[2] Shulman GL, Fiez JA, Corbetta M, Buckner RL, Miezin FM, Raichle ME (1997). Common blood flow changes across visual tasks: II. Decreases in cerebral cortex. J Cogn Neurosci.

[3] Greicius MD, Krasnow B, Reiss AL, Menon V (2003). Functional connectivity in the resting brain: a network analysis of the default mode hypothesis. Proc Natl Acad Sci.

[4] Fox MD, Snyder AZ, Vincent JL, Corbetta M, Essen DCV, Raichle ME (2005). The human brain is intrinsically organized into dynamic, anticorrelated functional networks. Proc Natl Acad Sci USA.

[5] Passow S, Specht K, Adamsen TC, Biermann M, Brekke N, Craven AR (2015). A close link between metabolic activity and functional connectivity in the resting human brain. EJNMMI Phys.

[6] Watabe T, Shimosegawa E, Kato H, Isohashi K, Ishibashi M, Hatazawa J (2014). CBF/CBV maps in normal volunteers studied with ^15^O PET: a possible index of cerebral perfusion pressure. Neurosci Bull.

[7] Matsumoto K, Kitamura K, Mizuta T, Tanaka K, Yamamoto S, Sakamoto S (2006). Performance characteristics of a new 3-dimensional continuous-emission and spiral-transmission high-sensitivity and high-resolution PET camera evaluated with the NEMA NU 2-2001 standard. J Nucl Med Off Publ Soc Nucl Med.

[8] Ibaraki M, Miura S, Shimosegawa E, Sugawara S, Mizuta T, Ishikawa A (2008). Quantification of cerebral blood flow and oxygen metabolism with 3-dimensional PET and ^15^O: validation by comparison with 2-dimensional PET. J Nucl Med Off Publ Soc Nucl Med.

[9] Mintun MA, Raichle ME, Martin WR, Herscovitch P (1984). Brain oxygen utilization measured with O-15 radiotracers and positron emission tomography. J Nucl Med Off Publ Soc Nucl Med.

[10] Raichle ME, Martin WR, Herscovitch P, Mintun MA, Markham J (1983). Brain blood flow measured with intravenous H_2_(^15^)O. II. Implementation and validation. J Nucl Med Off Publ Soc Nucl Med.

[11] Iida H, Jones T, Miura S (1993). Modeling approach to eliminate the need to separate arterial plasma in oxygen-15 inhalation positron emission tomography. J Nucl Med Off Publ Soc Nucl Med.

[12] Hatazawa J, Fujita H, Kanno I, Satoh T, Iida H, Miura S (1995). Regional cerebral blood flow, blood volume, oxygen extraction fraction, and oxygen utilization rate in normal volunteers measured by the autoradiographic technique and the single breath inhalation method. Ann Nucl Med.

[13] Lammertsma AA, Jones T (1983). Correction for the presence of intravascular oxygen-15 in the steady-state technique for measuring regional oxygen extraction ratio in the brain: 1. Description of the method. J Cereb Blood Flow Metab Off J Int Soc Cereb Blood Flow Metab.

[14] Kanno I, Iida H, Miura S, Murakami M, Takahashi K, Sasaki H (1987). A system for cerebral blood flow measurement using an H_2_^15^O autoradiographic method and positron emission tomography. J Cereb Blood Flow Metab Off J Int Soc Cereb Blood Flow Metab.

[15] Iida H, Kanno I, Miura S, Murakami M, Takahashi K, Uemura K (1986). Error analysis of a quantitative cerebral blood flow measurement using H_2_(^15^)O autoradiography and positron emission tomography, with respect to the dispersion of the input function. J Cereb Blood Flow Metab Off J Int Soc Cereb Blood Flow Metab.

[16] Fair DA, Cohen AL, Dosenbach NU, Church JA, Miezin FM, Barch DM (2008). The maturing architecture of the brain’s default network. Proc Natl Acad Sci.

[17] Di X, Biswal BB (2012). Metabolic Brain Covariant Networks as Revealed by FDG-PET with Reference to Resting-State fMRI Networks. Brain Connect.

[18] Xia M, Wang J, He Y (2013). BrainNet viewer: a network visualization tool for human brain connectomics. PLoS One.

[19] Fox PT, Raichle ME, Mintun MA, Dence C (1988). Nonoxidative glucose consumption during focal physiologic neural activity. Science.

[20] Paulson OB, Hasselbalch SG, Rostrup E, Knudsen GM, Pelligrino D (2010). Cerebral blood flow response to functional activation. J Cereb Blood Flow Metab.

[21] Kameyama M, Murakami K, Jinzaki M (2016). Comparison of [^15^O] H_2_O positron emission tomography and functional magnetic resonance imaging in activation studies. World J Nucl Med.

[22] Perlmutter JS, Powers WJ, Herscovitch P, Fox PT, Raichle ME (1987). Regional asymmetries of cerebral blood flow, blood volume, and oxygen utilization and extraction in normal subjects. J Cereb Blood Flow Metab.

